# Retrospective case series describing the efficacy, safety and cost-effectiveness of a vial-sharing programme for canakinumab treatment for paediatric patients with cryopyrin-associated periodic syndrome

**DOI:** 10.1186/s12969-019-0335-4

**Published:** 2019-07-08

**Authors:** Abdulkadir A. Elmi, Karen Wynne, Iek L. Cheng, Despina Eleftheriou, Helen J. Lachmann, Philip N. Hawkins, Paul Brogan

**Affiliations:** 1 Infection Inflammation and Rheumatology Section, University College London Institute of Child Health, 30 Guilford St, London, WC1N1EH UK; 2 Department of Paediatric Rheumatology, Great Ormond Street Hospital NHS Foundation Trust, London, UK; 3 University College London National Amyloidosis Centre, London, UK

**Keywords:** Canakinumab, Cryopyrin-associated periodic syndrome (CAPS), Vial-sharing, Child, Paediatric

## Abstract

**Background:**

Cryopyrin-associated periodic syndrome (CAPS) is a rare autoinflammatory disease, caused by gain of function mutation in *NLRP3* resulting in excess production of interleukin-1 (IL-1). Canakinumab is a human monoclonal antibody against Interleukin-1 beta (IL-1β), licensed for the treatment of CAPS**.** The objective of the study was to describe the feasibility and cost-effectiveness of a canakinumab vial-sharing programme for paediatric patients with CAPS.

**Method:**

Retrospective case series and clinical service description of a national specially commissioned CAPS clinic at Great Ormond Street Hospital (GOSH). Effectiveness was assessed using a CAPS disease activity score (DAS) and serum amyloid A protein (SAA). Adverse events were collected to determine safety. The number of canakinumab vials saved was considered when investigating the cost-effectiveness of vial-sharing.

**Results:**

Nineteen/20 (95%) of our paediatric patients achieved minimally active clinical disease activity with canakinumab monotherapy; and 75% achieved both minimally active clinical disease and serological remission using a pre-specified definition based on the CAPS DAS and SAA level. Canakinumab was well tolerated, with only one child developing an infection requiring hospitalisation during the study. Canakinumab vial sharing resulted in 117 vials of canakinumab saved over a 24-month period, equating to a direct drug-related cost saving of £1,385,821, and a conservative estimated 5-year cost-saving of £3,464,552.50.

**Conclusion:**

We provide further evidence for the effectiveness and safety of canakinumab in children with CAPS, and highlight the cost-effectiveness of a vial-sharing programme for this high cost medicine. We suggest that this could have important implications for the delivery of other high cost medicines used in paediatric practice.

**Electronic supplementary material:**

The online version of this article (10.1186/s12969-019-0335-4) contains supplementary material, which is available to authorized users.

## Background

Cryopyrin-associated periodic syndromes (CAPS) are a group of rare, monogenic, autoinflammatory diseases caused by a gain of function mutation in NLR family pyrin domain containing 3 (*NLRP3*), which results in excess production of interleukin-1 beta (IL-1β) [1]. This triggers disabling multi-system inflammation from birth, with systemic and organ-specific complications of varying severity, and a long-term risk of reactive, AA amyloidosis. Three main phenotypes are recognised on a continuum of severity [1–3]. The most severe form of CAPS is Chronic Infantile Neurological Cutaneous Articular syndrome (CINCA), associated with severe systemic inflammation from early in life with urticarial rash, central nervous system inflammation, neurological impairment, various inflammatory ophthalmological sequelae, severe musculoskeletal morbidity, and impairment of growth and development. Muckle-Wells syndrome (MWS) is of intermediate severity, with skin rash, periodic fever, and risk of sensorineural hearing loss. The mildest form is familial cold-induced autoinflammatory syndrome (FCAS), typified by cold-induced urticaria, myalgia, malaise and fatigue. There is a surprising lack of genotype-phenotype correlation and little reliable prediction of disease severity based on specific *NLRP3* mutations [4].

Canakinumab is a high affinity IgG1 monoclonal antibody against interleukin 1β which first received a licence for the treatment of CAPS in 2009. Canakinumab is currently licensed for CAPS in patients from the age of 2 years upwards, and is highly efficacious in this context, resulting in rapid relief of symptoms and normalisation of acute-phase responses in most patients with CAPS [5].

We previously described the paediatric component of a new nationally commissioned highly specialised service for patients in England with CAPS, adding real-world experience regarding the use of canakinumab in a paediatric setting, with outcome data subsequently updated and published online every 2 years [3] (https://www.gosh.nhs.uk/conditions-and-treatments/clinical-outcomes/rheumatology-clinical-outcomes). Over the first 2 years of this clinic, it became increasingly apparent that dosing young children using adult sized vials (150 mg per vial) resulted in considerable drug wastage, since paediatric doses are based on weight, typically starting at 2 mg/kg subcutaneously every 8 weeks for children 10 kg upwards. To address this issue, we introduced a vial-sharing programme for children receiving canakinumab for CAPS at Great Ormond Street Hospital NHS Foundation Trust (GOSH). The purpose of this study was to describe the impact of this innovation, and describe generic lessons learned that could have implications when considering similar cost saving initiatives for other high-cost medicines.

## Materials and methods

### Aims and objectives

The aim of the project was to report the clinical and cost-effectiveness of a vial-sharing programme for canakinumab treatment in paediatric patients with CAPS at the Nationally Specialist Commissioned CAPS clinic at Great Ormond Street Hospital (GOSH) NHS Foundation Trust.

The objectives of this project were to:

1. Describe the demographics of the expanding cohort of paediatric patients attending a national specialist clinic for CAPS at GOSH.
2. Describe the therapeutic effectiveness of canakinumab, using a standardised disease activity score and a sensitive serological marker of inflammation (Serum amyloid A).
3. Describe safety based on adverse events.
4. Assess the cost of canakinumab in this context, and the economic impact of a systematic vial-sharing program at GOSH, including the development of a spreadsheet package to facilitate this analysis retrospectively, and to model future cost savings based on projected growth of the clinical service over the next 5 years.

### Design and patients

This was a single-centre, retrospective, observational study of paediatric CAPS patients receiving canakinumab between November 1st 2015, and October 31st 2017 at the national specially commissioned CAPS Clinic at GOSH. This is the only specialised paediatric service across the United Kingdom for patients with this ultra-rare autosomal dominant genetic disease. Since this was a retrospective case notes review with presentation of fully anonymised data, written patient/parental consent was not required. Patients with CAPS were eligible for canakinumab treatment if they were ≥ 2 years of age; had clinical features of active CAPS requiring medical intervention; and had a confirmed mutation in the NLRP3 gene; or had characteristic clinical features of CAPS if they were *NLRP3* mutation negative [6]. Exclusion criteria were evidence of a pre-existing underlying infection or any other significant medical condition that would preclude treatment with canakinumab. Research and ethical approval for this retrospective study was granted from the Joint Research and Development Office of Great Ormond Street Hospital, in accordance with UK law (reference number 16IR47).

### Assessment of CAPS disease activity

A standardised approach was used to monitor CAPS disease activity, as described previously in this clinic [3]. In brief, this consisted of standardised documentation of clinical disease activity using the CAPS disease activity score (DAS) (please see Additional file 1). The DAS depicts disease activity using ten symptoms/signs; absence of disease activity was defined as a score of 0/20; minimal disease activity was depicted by a score ≤ 3/20; and a maximum score of 20 indicated maximal disease activity [3]. Serological response was monitored using serum amyloid A (SAA, reference range < 10 mg/L). Using these indices, minimally active disease was defined as absent or minimal disease activity (DAS ≤3/20 with no item scoring as severe) and normal markers of inflammation (SAA < 10 mg/L). The CAPS DAS and SAA (plus other routine investigations: full blood count, renal and liver function; and C-reactive protein) were assessed as a minimum every 2 months from commencement of treatment.

### Canakinumab vial sharing and assessment of cost savings

Vial sharing was instituted as part of the clinical CAPS service at GOSH in November 2015. The paediatric CAPS clinic occurs monthly, and all patients attend GOSH for clinical assessment, laboratory investigation and dosing. One important advantage of centralisation of care for this very rare disease is that it can facilitate vial sharing of canakinumab: individual patient doses (typically, 10 patients per clinic) are made up centrally in the pharmacy aseptic unit using the required number of vials to generate these doses resulting in minimum wastage.

For a full description of how canakinumab is reconstituted, we refer the reader to the summary of product characteristics, available at: (https://www.ema.europa.eu/en/documents/product-information/ilaris-epar-product-information_en.pdf). In brief, canakinumab comes in vials containing 150 mg powder for reconstitution with 1 ml of clear and colourless solvent. After reconstitution, it has a 24-h refrigerated (4–8 °C) shelf-life. Exact doses for individual patients are taken from these reconstituted vials aseptically in pharmacy, for subcutaneous injection into the patient on the ward on the same day as clinic.

The cost of canakinumab (before and after value added tax, VAT) was derived from the National Institute for Health and Care Excellence [Accessed April 21, 2017] (https://www.nice.org.uk/advice/esnm23/chapter/key-points-from-the-evidence). From that, direct cost savings were calculated by subtracting the cost of actual number of vials used from the number of vials that would have been required if one (or more, if under exceptional circumstances the canakinumab dose exceeded 150 mg) vials had been allocated to each patient. Pharmacy costs required to deliver central drug preparation were also taken into account when calculating the final cost saving. Using this approach, the actual cost saving was calculated for a 24 month period starting from November 1st, 2015 (i.e. spanning 24 clinics). Conservative projected cost savings based on this were extrapolated over the next 5 years.

### Data handling and statistics

The project utilised descriptive statistics for categorical data, and summarised as percentage unless otherwise stated; numeric data were expressed as median and range. The differences in categorical variables were investigated using Fisher’s exact test with significance set at a two-tailed *p*-value of less than 0.05. Fisher’s exact test was calculated using a freely available online software (https://www.graphpad.com/quickcalcs/contingency1.cfm). Microsoft Excel 2016 was used to produce a spreadsheet used to investigate the cost savings of vial-sharing to produce graphical outputs.

## Results

### Baseline clinical features

Since its inception in 2011, the paediatric CAPS service at GOSH has expanded every year, from 14 patients in 2013 to 39 patients who had attended the children’s CAPS service by the end of 2016. At the time of this study, the number of patients currently treated by the service was 28 (11 having been successfully transitioned to adult care). Of these, 20/28 patients who were receiving canakinumab were included in this study (Table 1). The remaining eight patients were excluded due to receiving anakinra treatment (*n* = 3) or awaiting treatment to be commenced (*n* = 5).

**Table 1:** Summary of patients attending the national paediatric cryopyrin associated periodic fever syndrome service

Total number of patients who ever attended the GOSH CAPS service		39
Total number of patients included for study		20
Total number of patients with negative *NLRP3* mutation		4
Total number of doses of canakinumab ever received per patient	Median	14
	Range	7–41 doses
Age	Median	9 years
	Range	4–14 years
Gender	Male	10
	Female	10

*CAPS* Cryopyrin associated periodic fever syndrome, *GOSH* Great Ormond Street Hospital

### Response to canakinumab

**Fig. 1:**
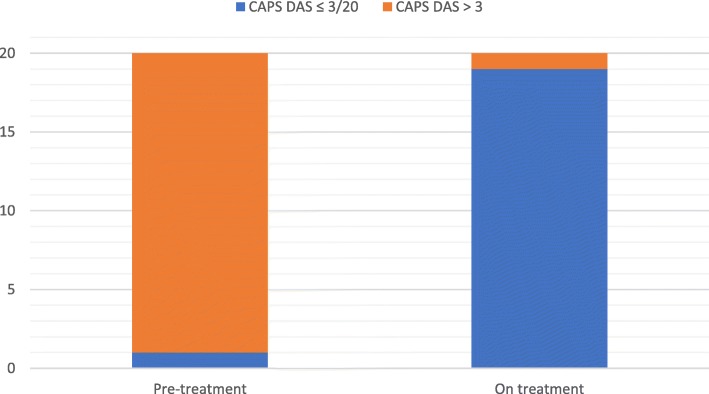
Cryopyrin associated periodic syndrome disease activity score pre-and post canakinumab. *The difference between CAPS DAS scores before and after canakinumab treatment was highly significant (P < 0.0001).* A standardised approach was used to monitor CAPS disease activity, as described previously in this clinic [3]. In brief, this consisted of standardised documentation of clinical disease activity using the CAPS disease activity score (DAS) (please see Additional file 1). The DAS depicts disease activity using ten symptoms/signs; absence of disease activity was defined as a score of 0/20; minimal disease activity was depicted by a score ≤ 3/20; and a maximum score of 20 indicated maximal disease activity. The figure shows that before commencing canakinumab treatment, the median CAPS DAS was 6.5/20 (range 0–11/20), with 95% of patients scoring > 3/20 (Fig. 1)

**Fig. 2:**
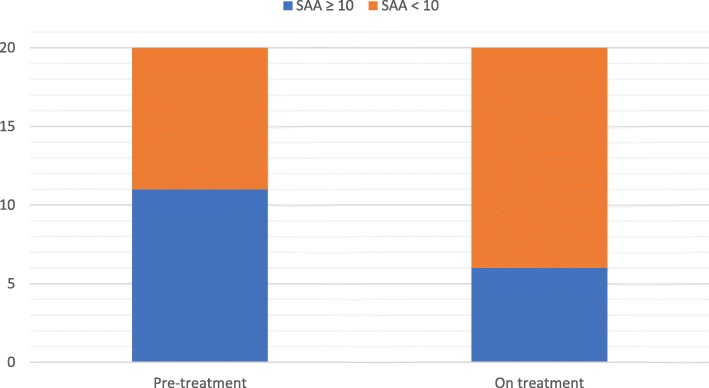
Serum Amyloid A levels score pre-and post canakinumab. Serological response was monitored using serum amyloid A (SAA, reference range < 10 mg/L). The figure shows that the median SAA before treatment was 34.8 mg/L (range 0–497; reference range [RR] less than 10 mg/L); and SAA was raised (higher than upper RR) in 11/20 (55%) patients (Fig. 2)

**Fig. 3:**
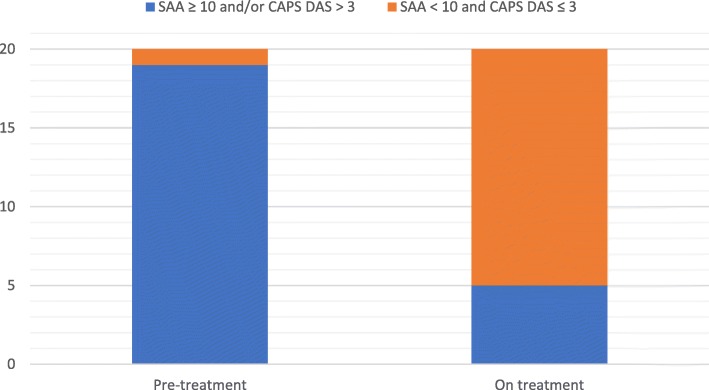
CAPS disease activity score and Serum Amyloid A levels score pre-and post canakinumab. The difference between CAPS DAS and SAA scores before and after canakinumab was significant (*P* < 0.0001). Using these indices, minimally active disease was defined as absent or minimal disease activity (DAS ≤3/20 with no item scoring as severe) and normal markers of inflammation (SAA < 10 mg/L). The CAPS DAS and SAA (plus other routine investigations: full blood count, renal and liver function; and C-reactive protein) were assessed as a minimum every 2 months from commencement of treatment. On canakinumab, 19/20 (95%) patients had a CAPS DAS ≤ 3 (Fig. 1); and 14/20 (70%) patients had a SAA of < 10 mg/L (Fig. 2). After canakinumab treatment, 15/20 (75%) patients had CAPS DAS ≤ 3 and a SAA < 10 mg/L (Fig. 3)

Before commencing canakinumab treatment, the median CAPS DAS was 6.5/20 (range 0–11/20), with 95% of patients scoring > 3/20 (Fig. 1). The median SAA before treatment was 34.8 mg/L (range 0–497; reference range [RR] less than 10 mg/L); and SAA was raised (higher than upper RR) in 11/20 (55%) patients (Fig. 2). On canakinumab, 19/20 (95%) patients had a CAPS DAS ≤ 3 (Fig. 1); and 14/20 (70%) patients had a SAA of < 10 mg/L (Fig. 2). After canakinumab treatment, 15/20 (75%) patients had CAPS DAS ≤ 3 and a SAA < 10 mg/L (Fig. 3).

### Safety of canakinumab

Seven patients developed infections after a median treatment period of 16 months (range 2–33). These were: bacterial tracheitis (*n* = 1); presumed viral upper respiratory tract infection (*n* = 1); lower respiratory tract infection, no organism documented (*n* = 3); uncomplicated varicella zoster virus infection (*n* = 1); presumed viral gastroenteritis (*n* = 1). Only one infection episode resulted in hospitalisation for intravenous antibiotics (the case of bacterial tracheitis, no organism isolated, but prompt response to antibiotic therapy). There were no other adverse effects documented; in particular, there were no injection site reactions reported.

### Savings with Canakinumab vial-sharing

Table 2 summarises the actual direct drug cost saving over a 24-month period starting from November 1st 2015. The total cost of one vial of canakinumab before value added tax (VAT), as stated by both the National Institute for Health and Care Excellence (NICE) and British National Formulary (BNF), is £9927.80 (https://www.nice.org.uk/advice/esnm23/chapter/key-points-from-the-evidence). The cost for a single vial after VAT is £11,913.

**Table 2:** Actual (2015–2017) and 5-year projected cost savings from canakinumab vial-sharing

Total number of vials used if no vial sharing^a^	268
Total number of vials used with vial sharing^a^	151
Total number of vials saved over 24 months^a^	117
Cost of one 150 mg canakinumab vial (excluding VAT)	£9927.80
Cost of one 150 mg canakinumab vial (including VAT)	£11,913
Total drug cost without vial sharing^b^	£3,192,684
Total drug cost with vial sharing^b^	£1,798,863
Cost saving from vial sharing over 24 months (Including preparation cost)	£1,385,821
Projected net cost saving over the next five years^c^ (Including preparation cost)	£3,464,552.50

^a^1.11.15–31.10.17, spanning 24 clinics; ^b^Total costs includes VAT, since canakinumab administered in hospital; ^c^assuming no growth in patient numbers, based on net cost saving 2015–2017

Vial sharing resulted in an actual direct drug cost saving of £1,393,821 over a 24-month period. Subtracting costs associated with central pharmacy canakinumab preparation (£4000 per annum), the net annual cost saving was £1,385,821, providing a 5-year projected net cost saving of approximately £3.5 million (assuming no further growth in patient numbers).

## Discussion

Controlled and uncontrolled studies unequivocally demonstrate that canakinumab is a highly effective and safe treatment for children and adults with CAPS [3], as reinforced by the data presented here. In the context of a national specially commissioned paediatric CAPS clinical service, 95% of our paediatric patients achieved minimally active clinical disease activity; and 75% achieved both minimally active clinical disease and serological remission using a pre-specified definition based on the CAPS DAS and SAA level. Moreover, canakinumab was well tolerated, with only one child developing an infection requiring hospitalisation. Thus, canakinumab is effective and safe in children with CAPS. Canakinumab, however, is expensive costing approximately £12,000 per vial, and many children require less than one vial per dose. To address this, we designed our clinical service to facilitate vial sharing having observed considerable drug wastage in the first 4 years of this specialist clinic.

We first introduced this vial sharing programme in November 2015, and explored the relative merits but also potential downsides of such an approach over the next 24 months. A total of 117 vials were saved over a 24-month period (24 clinics), providing a cost saving of approximately £1.39 million (which includes VAT, since in the UK this is added to the cost of medicines administered to patients in hospital), after subtracting the modest cost associated with central pharmacy preparation of individual patient doses. To provide a conservative estimate of the projected 5-year cost savings, we assumed that the number of patients treated and number of vials used would remain constant, which provided an estimate of a £3.5 m saving over 5 years. Given that the paediatric CAPS service has thus far grown yearly since 2011, the actual cost saving may turn out to be considerably higher. However, we acknowledge that this is a crude 5-year estimate that could be affected by changes in patient doses over time that may influence number of vials saved, and other market forces such as changes in the price of canakinumab (although we are not aware of any plans to change the price of canakinumab in the foreseeable future).

This model could have implications for the use of other high-cost children’s medicines. Our simple innovation was entirely dependent on running the clinic on the same day of the month for all CAPS patients, which enabled us to a priori calculate the total clinic dose required 2 weeks in advance. Centralised pharmacy preparation of individual doses used a much reduced number of canakinumab vials and only required very modest investment (£4000 per annum). We suggest that this innovation will help secure resilience for paediatric CAPS patient access to this high cost medicine for years to come, whilst generating considerable savings for the NHS for the foreseeable future. It also improves patient safety by reducing the likelihood of medication errors since drug is reconstituted aseptically centrally by a dedicated hospital pharmacist. Furthermore, it improves the patient experience by reducing their length of visit as doses are prepared ahead of their visits.

General points to consider when contemplating vial-sharing for other high cost medicines are summarised in Table 3. Whilst there are clear benefits of centralisation of highly specialist clinical services, such as (importantly) accrual of unique clinical expertise as a direct consequence of high throughput of patients with an exceptionally rare disease; and the highlighted economic benefits we describe here in; two important downsides are noteworthy. First and foremost, patients have to travel, sometimes from afar, to the centre of expertise. This is on the whole manageable for patients with CAPS, since dosing is every 8 weeks, and additionally centralises and consolidates clinical expertise for this ultra-rare disease. Secondly, it is not possible to make real-time dose changes on the day of the clinic since the canakinumab is prescribed and prepared in advance. In practice this is not a major issue for CAPS patients, because canakinumab has a very long half-life (approximately 28 days), and therefore dose alterations can safely occur at the next clinic visit (Table 3). These latter 2 points are however important to consider when contemplating vial sharing for other high cost medicines.

**Table 3:** Suggested characteristics of a successful vial sharing programme for high cost medicines

Characteristic	Comments
Well defined patient population	Published diagnostic criteria for CAPS are available, and genetic testing for *NLRP3* mutations is available.
Expensive medicine	The cost of one vial of canakinumab is £11,913.
Risk of drug wastage in paediatric population	Example: A 10 kg baby with CAPS would have a typical starting dose of 20 mg. This means that without vial sharing 130 mg of the vial would be wasted.
Patients attend single centre on same day as dose	This service only applies to NHS England patients; some travel long distances every 8 weeks.
Dosage interval acceptable for regular travel to single centre	Patients receive canakinumab every 8 weeks, minimising impact on families of regular travel to the national CAPS clinic.
Sufficient numbers of patients to make vial sharing feasible	Whilst CAPS as an exceptionally rare disease, centralisation of the national clinic provides sufficient patient numbers to ensure that vial sharing becomes a feasible and cost-effective exercise.
Availability of a pharmacy aseptic unit	Individual doses need to be made under aseptic conditions to allow vial sharing. Centres will require an aseptic unit with the capacity to fulfil the demand to implement this innovative practice.
Nationally commissioned service in place	National funding arrangement agreed from NHS England for the use of canakinumab in patients with CAPS. This was a key aspect facilitating centralisation of care, and thus a viable vial sharing programme.
Acute “real-time” changes in drug dose not critical	Changes in canakinumab dose cannot be made on the day of clinic since this is prescribed 2 weeks in advance. Dosage alteration therefore occurs 8 weeks later at the next clinic visit.

## Conclusion

In conclusion, canakinumab is highly effective for paediatric patients with CAPS, and demonstrates excellent safety and tolerability. We demonstrate the cost-effectiveness of a canakinumab vial-sharing programme, which inarguably offsets some of the high cost associated with this treatment. We suggest that this approach could be applied to other high-cost medicines used in paediatric practice.

## Additional file

Additional file 1: Canakinumab clinical assessment pro forma. (DOCX 63 kb)

## Data Availability

Availability of data and material: this report contains anonymised data and material collected from retrospective analyses of medical records stored as part of routine clinical care to preserve patient anonymity.

## Abbreviations

BNF: British National Formulary
CAPS: Cryopyrin-associated periodic syndrome
CINCA: Chronic infantile cutaneous neurological articular syndrome
DAS: Disease activity score
FCAS: Familial cold-induced autoinflammatory syndrome
GOSH: Great Ormond Street Hospital
IL-1β: Interleukin-1 beta
MWS: Muckle-Wells syndrome
NHS: National Health Service
NICE: National Institute for Health and Care Excellence
*NLRP3*: NLR family pyrin domain containing 3
R&D: Research and Development
SAA: Serum Amyloid A
