# Supplementary figures and images for: Retrospective case series describing the efficacy, safety and cost-effectiveness of a vial-sharing programme for canakinumab treatment for paediatric patients with cryopyrin-associated periodic syndrome
Source: Pediatr Rheumatol Online J. 2019 Jul 8;17:36. doi: 10.1186/s12969-019-0335-4 (PMC6615159; doi:10.1186/s12969-019-0335-4)

### Appendix A- Canakinumab clinical assessment pro forma


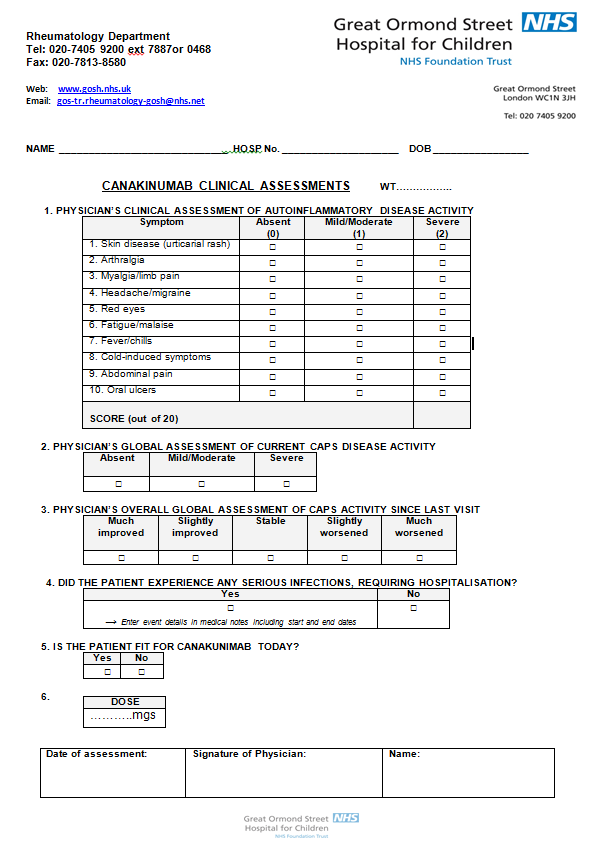

Supplement: Supplementary file 1 — Canakinumab clinical assessment pro forma. (DOCX 63 kb) [file 12969_2019_335_MOESM1_ESM.docx]
